# Investigating the impact of a community home-based care on mental health and anti-retroviral therapy adherence in people living with HIV in Nepal: a community intervention study

**DOI:** 10.1186/s12879-018-3170-1

**Published:** 2018-06-07

**Authors:** Khem N. Pokhrel, Vidya D. Sharma, Kalpana G. Pokhrel, Sanjeev R. Neupane, Linda B. Mlunde, Krishna C. Poudel, Masamine Jimba

**Affiliations:** 1 Health Research and Social Development Forum Nepal, P.O. Box 24133, Thapathali, Kathmandu, Nepal; 2 Department of Psychiatry and Mental Health, Institute of Medicine, Tribhuwan University, Kathmandu, Nepal; 3 Integrated Development Foundation Nepal, Kathmandu, Nepal; 4 Integrated Development Foundation Nepal, Kathmandu, Nepal; 5 Management and Development for Health, P.O. Box 79810, Dar es Salaam, Tanzania; 6 Department of Public Health Policy, School of Public Health and Health Sciences, University of Massachussets Amherst, Boston, USA; 7 Department of Community and Global Health, Graduate School of Medicine, The University of Tokyo, Bunkyo City, Tokyo, Japan

**Keywords:** Mental health, Substance use, HIV, Anti-retroviral therapy, Nepal

## Abstract

**Background:**

HIV-positive people often experience mental health disorders and engage in substance use when the disease progresses. In resource limited settings, mental health services are not integrated into HIV services. In Nepal, HIV-positive people do receive psychosocial support and other basic health care services from a community home-based care intervention; however, the effects of the intervention on health outcomes is not yet known. Therefore, we examined the impact of the intervention on mental health and antiretroviral therapy (ART) adherence.

**Methods:**

We conducted an intervention study to identify the effects of a community home-based care intervention on mental health disorders, substance use, and non-adherence to ART among HIV-positive people in Nepal from March to August 2015. In total, 344 participated in the intervention and another 338 were in the control group. The intervention was comprised of home-based psychosocial support and peer counseling, adherence support, basic health care, and referral services. We measured the participants’ depression, anxiety, stress, substance use, and non-adherence to ART. We applied a generalized estimating equation to examine the effects of intervention on health outcomes.

**Results:**

The intervention had positive effects in reducing depressive symptoms [Adjusted Odds Ratio (AOR) = 0.44, *p* < 0.001)], anxiety (AOR = 0.54, *p* = 0.014), stress (β = − 3.98, *p* < 0.001), substance use (AOR = 0.51, *p* = 0.005), and non-adherence to ART (AOR = 0.62, *p* = 0.025) among its participants at six-month follow-up.

**Conclusions:**

The intervention was effective in reducing mental health disorders, substance use, and non-adherence to ART among HIV-positive people. Community home-based care intervention can be applied in resource limited setting to improve the mental health of the HIV-positive people. Such intervention should be targeted to include more HIV-positive people in order to improve their ART adherence.

**Trial registration:**

ClinicalTrials.gov ID: NCT03505866, Released Date: April 20, 2018.

## Background

HIV-positive people often endure poor mental health status and have inadequate access to health services [1]. Such mental health status may become chronic when they do not have access to appropriate services for screening and treatment [2, 3]. Specifically, many of them frequently experience various mental health disorders and engage in substance use when the HIV infection progresses [4–7]. Consequently, such conditions negatively affect their adherence to antiretroviral therapy (ART) [8, 9].

Community-based psychosocial support may be an integral part of care and support services for HIV-positive people in low- and middle-income countries (LMICs). To address their need for psychosocial support, the World Health Organization (WHO) provided guidelines to improve these individuals’ mental health [10]. The guidelines recommend that HIV-positive people, their families, and their caregivers receive psychosocial support at the family and community level. Such psychosocial support may contribute to improvements in the health and treatment outcomes of HIV-positive people [11].

Various community-based psychosocial support programs have improved health outcomes of HIV-positive people in LMICs [12, 13]. In Peru, for instance, HIV-positive people improved their mental health status when they received emotional and economic support from volunteer health workers at health facilities [14]. Similarly, community-based support improved ART adherence in Brazil [15]. However, these services were mostly facility-based and did not involve the home as a place for care and support; the efficiency and impact of community home-based care may be even greater in comparison [16].

Only limited data are available about the roles of community home-based care programs in improving health outcomes of HIV-positive people. In Vietnam, HIV-positive people improved their quality of life when they received peer support at their homes [17]. Home-based psychosocial support by field officers also improved ART adherence in Uganda [18], and home-based care delivered through nurses improved ART adherence in China [19].

However, looking at the WHO guidelines for community home-based care, little is known about the effects of essential components of care for HIV-positive people [16]. If all the essential components of care recommended by these guidelines (i.e., psychosocial support and counseling, ART adherence support and counseling, peer counseling, basic health care for opportunistic infections and sexually transmitted infection, and referral linkage of HIV-positive people and their families with health service providers) were to be included in a community home-based care intervention, it would be more effective.

Nepal has promised to provide basic mental health services at the community level. Such services, however, are still inadequate there [20]. Additionally, mental health services are not integrated into HIV programs in the country. However, these services are necessary because HIV-positive people have high prevalence of mental health disorders and substance use in Nepal. Among them, 25% experience depressive symptoms and 15% engage in substance use [21].

To fill this gap, a community home-based care intervention in Nepal provides HIV-positive people with care and support services. The program team is comprised of a community health worker, a trained HIV-positive person, and a social worker. The team conducts a monthly home-visit to provide psychosocial support and peer counseling, ART adherence support and counseling, basic health care, and referral for further care [22]. The program is unique, voluntary, and need-based. It includes HIV-positive people for peer counseling, links HIV-positive people to health service providers, and reduces physician and transportation cost.

Since the inception of community home-based care intervention, our study is the first to examine the effects that this program has had in improving mental health outcomes. We first examined the effects of a community home-based care program in reducing depressive symptoms, anxiety, stress, and substance use among HIV-positive people after 6 months. Second, we examined its effects in reducing the participants’ non-adherence to ART in Nepal.

## Methods

### Study design and settings

This community-based intervention study was conducted in Nepal from March to August 2015. About 28 million people live in the country, including 39,249 HIV-positive people as of 2015 [23]. Of them, 11,089 received ART in the same year. The intervention participants were evaluated at the beginning of the study and then followed up with after 6 months. The control group received regular services whereas the intervention participants were enrolled in community home-based care intervention.

### Sampling strategy and participants

#### Sample size

Sample size was calculated using G*Power software. Prevalence of non-adherence to ART was taken as 21% among HIV-positive people who received community-based psychosocial support as a reference [14]. We considered 10% difference in outcome as non-adherence to ART among HIV-positive people who received psychosocial support from those who did not receive the support [15]. Assuming *P*-value for significant of less than 0.05 (two-tailed) and the power of 0.80, the calculated sample size was 638 (intervention: 319 and control: 319). A total of 682 (intervention: 344 and control: 338) participants were recruited in this study out of 720 reached.

#### Participants

A convenience sampling method was adopted to select the study districts and the participants. Out of 23 districts in Nepal with high prevalence of HIV, 12 districts had a community home-based care program, and the remaining 11 districts had mutual support groups of HIV-positive people [24]. Mutual support groups are informal group among HIV-positive people who support each other in the group for routine HIV services such as helping HIV-positive people to approach ART centers and supporting those who were excluded in the community. The support group is an informal association of HIV-positive people.

Participants of the intervention group were purposively selected out of those who had voluntarily agreed to enroll in a community home-based care program from three districts: Kathmandu, Kaski, and Bake. Participants for the control group were purposively selected from the two districts Palpa and Nuwakot out of the 11 that had mutual support groups of HIV-positive people. In total, 682 (intervention: 344 and control: 338) participants were recruited in this study. The inclusion criteria for study participants were as follows:

- Intervention group
  The intervention group included HIV-positive people who a) had been diagnosed as HIV positive within 5 years of baseline data collection, b) had been receiving ART for at least 1 year, c) had been referred by an ART center or a voluntary counseling and testing (VCT) center, and d) had voluntarily agreed to enroll in a community home-based care program.
- Control group
  The control group included HIV-positive people who received routine ART medication and support services and who a) had been diagnosed as HIV positive within 5 years within the baseline data collection, b) were accessible through their mutual support groups, c) were living in the districts that did not have a community home-based care program, and d) had been receiving ART for at least 1 year. Among them, those who were below 18 years of age at the time of data collection were excluded from the study.

### Intervention

The intervention group received services from a comprehensively designed community home-based care program. The intervention was started on March 1, 2015, and was completed on August 31, 2015. The program was comprised of psychosocial support and peer counseling, ART adherence support and counseling, basic health care, and referral for further care. The support team was comprised of a community health worker, a trained HIV-positive person, and a social worker. The team received 5 days of training from a community home-based care network to improve their skills on home-based palliative care and psychosocial support. The program team performed monthly home-visits and spent at least 2 h at the homes of HIV-positive people to provide services.

In each home-visit, the program team performed comprehensive care and support services for the intervention group. Regarding psychosocial support, the trained team provided individual counseling, family counseling, and spousal counseling. A trained HIV-positive person was involved in each team to provide peer counseling and support. The participants received counseling on coping skills for the side effects of treatment, stress management, and self-care skills for physical symptoms. Substance users also received addiction and harm reduction counseling from a HIV-positive peer.

The intervention group received basic health care for opportunistic infections and STIs from a community health worker. The participants also received ART adherence support and counseling. To prevent the participants from missing pills, the team carefully checked the number of pills they had taken in the past month. A social worker in the team also helped them to manage their social issues and encouraged them to engage in HIV support services. For severe and unmanageable cases, the team referred them to further care in health facilities and for opioid substitution therapy.

### Measures

#### Mental health disorders

We used the Center for Epidemiologic Studies Depression (CESD) Scale to measure depressive symptoms that the participants had experienced in the past week [25]. The scale has 20 items and each item’s score ranges from 0 (for answers ranging from never to less than 1 day) to 3 (for answers including most of or all of the time (5–7 days)). The total score ranges from 0 to 60. Participants were considered to have depressive symptoms when they had scores of 16 or over [25]. This measure is widely used among HIV-positive people in various countries including Nepal [26]. In this study, its Cronbach’s alpha was 0.88.

We measured anxiety using Composite International Diagnostic Interview Short-Form (CIDI-SF) [27], which is a brief scale that has been applied in Nepal [28]. We used nine screening stem questions to identify the participants’ episodes of anxiety and difficulties in controlling their worries. We also asked about the presence of symptoms among those who answered in the affirmative to nine screening items. If participants had had more than three symptoms in the past month, we considered them as having anxiety.

We measured participants’ stress levels using Cohen’s Perceived Stress Scale (PSS). We asked participants whether they had had difficulties in coping with the difficulties in the past month using a 10-item scale. The items are in a Likert form and each item’s score ranges from “0” (never) to 4 (very often) and gives a total score range of 0–40 [29]. Having a higher score indicates a higher level of stress. This scale has already been tested and used in Nepal [30]. In this study, its Cronbach’s alpha was 0.82.

#### Substance use

We considered the participants as being a substance user if they reported use of an illicit substance at least once since their enrollment [31]. The list of illicit substances primarily included marijuana (cannabis), opium, non-prescriptive drugs, buprenorphine, diazepam, valium, amphetamines, and prescriptive drugs used without the advice of physician or psychiatrist.

#### ART non-adherence

We considered participants as non-adherent if they missed at least one dose in the past month of data collection by using adult AIDS clinical trial group (ACTG) questionnaires to assess the adherence [32].

#### Characteristics related to a community home-based care program

We collected the information about the use of services from a community home-based care program, using the questionnaires of standard operating procedures for these programs [22].

#### Sociodemographic and health related characteristics

The research team interviewed the participants about their age, gender, marital status, education, employment, and physical symptoms. Physical symptoms included opportunistic infections other than mental health complaints, such as cough, fever, diarrhea, herpes, etc..

### HIV-clinical staging

We obtained the information about the participants’ HIV-clinical staging from ART centers. We categorized the first and second clinical stages as early stage and the third and fourth stages as advanced stage [33].

### Procedure for data collection

We conducted a pretesting of questionnaires among 48 HIV-positive participants in Kathmandu Valley. The questionnaires were reviewed, analyzed and modified. At baseline, we interviewed 682 (intervention: 344 and control: 338) participants. We interviewed 655 (intervention: 329 and control: 326) participants at the follow-up. The follow-up of the participants was made through the service centers. We could not reach 27 participants (intervention: 12 and control: 15) at the follow-up out of total baseline participants. The participants could not be reached perhaps because of the termination of services by them, death, or another incident.

### Data analysis

We compared mental health disorders, substance use, and non-adherence to ART using chi-squared tests between the intervention and control groups. We used a generalized estimating equation (GEE) to examine the average population effects of the intervention on the outcome variables after 6 months of follow-up.

We developed five separate GEE models to examine the effects of a community home-based care program for five outcome variables. The variables included depressive symptoms, anxiety, stress levels, substance use, and non-adherence to ART. In all the models, we controlled for age, gender, education, marital status, employment status, HIV-clinical staging, and presence of physical symptoms. We set the statistical significance at *p* < 0.05 and used STATA 12 for data analyses.

### Ethical considerations

The Research Ethics Committee of the Graduate School of Medicine, the University of Tokyo and the Nepal Health Research Council (approval no. 1523/2015) approved this study. We required participants’ informed consent to participate in the study. Some participants who were unable to write their name provided their thumbprints in a designated consent form. We preserved the participants’ confidentiality of information and ensured that participation was voluntary. When we found participants with severe mental illness, we referred them to support agencies and health facilities. In addition, we provided a transportation cost of NRs. 150 ($1.41) to each participant as compensation.

## Results

### Baseline characteristics of intervention and control participants

Of the 682 participants, 344 (50.4%) enrolled in a community home-based care program and 338 (49.6%) were in mutual support groups. The mean age of participants in the intervention group was 36.3 years and 36.1 years in the control group was. In the intervention group, 53.1% were male and in the control group, 51% were male. At baseline, the intervention and control groups had similar characteristics for age (mean: 36.2 vs. 36.3, *p* = 0.730], gender (53.2% male vs. 50.6%, *p* = 0.149), having primary education or above (41.3% vs. 43.5, *p* = 0.559), marital status (84.0% unmarried vs. 78.4%, *p* = 0.061), and employment status (36.3% employed vs. 32.3%). Moreover, the intervention and control group had similar characteristics of being in advanced HIV-clinical stage (19.9% vs. 13.9%, *p* = 0.063) and having the presence of physical symptoms (23.5% vs. 19.2%, *p* = 0.170) (Table 1).

**Table 1:** Baseline characteristics of the intervention and control group

Characteristics	Intervention (*n* = 344) n (%)	Control (*n* = 338) n (%)	*P*-value
Age, mean (SD)	36.3 (8.1)	36.1 (8.0)	0.730
Gender			
Men	183 (53.2)	171 (50.6)	0.496
Women	161 (46.8)	167 (49.4)	
Education level			
No formal education	142 (41.3)	147 (43.5)	0.559
Primary level and above	202 (58.7)	191 (56.5)	
Marital status			
Married	289 (84.0)	265 (78.4)	0.061
Unmarried/single	55 (16.0)	73 (21.6)	
Employment			
Employed/self-employed	125 (36.3)	109 (32.3)	0.294
Unemployed	219 (63.7)	229 (67.7)	
HIV-clinical staging			
Early	266 (80.1)	284 (86.1)	0.063
Advanced	66 (19.9)	46 (13.9)	
Physical symptoms			
No	263 (76.5)	273 (80.8)	0.170
Yes	81 (23.5)	65 (19.2)	

### Mental health disorders, substance use, and ART non-adherence of intervention and control participants at baseline and follow-up

At baseline, of the 682 participants, 202 (29.6%) had depressive symptoms, 105 (15.4%) had anxiety, 125 (18.3%) used substances, and 185 (27.1%) were non-adherent. Among them, 48 (26.0%) were people who inject drugs. The intervention and control group had similar presence of depressive symptoms (29.7% vs. 29.6%, *p* = 0.937), anxiety (14.8% vs. 16.0%, *p* = 0.677), stress scores (mean: 16.5 vs. 15.7, *p* = 0.128), substance use (20.1% vs. 16.6%, *p* = 0.239), and non-adherence to ART (28.8, 25.4%, *p* = 0.959).

On the other hand, of the 655 participants at the six-month follow-up, 144 (22.0%) had depressive symptoms, 76 (11.6%) had anxiety, 81 (12.4%) used substances, and 125 (19.0%) were non-adherent. Compared with the control groups, the intervention groups had lower prevalence of having depressive symptoms (24.9% vs. 19.2%, *p* = 0.048), anxiety (14.4% vs. 8.8, *p* = 0.025), levels of stress (mean: 12.5 vs. 14.9, *p* < 0.001), substance use (15.3% vs. 9.4%, *p* = 0.021), and non-adherence to ART (23.9% vs. 14.3%, *p* = 0.002) at the six- month follow-up (Table 2).

**Table 2:** Mental health disorders, substance use, and non-adherence to ART at baseline and follow-up

Characteristics	Total	Intervention^a^	Control^b^	*P* value^c^
		*n* (%)	*n* (%)	
Depressive symptoms (yes)				
Baseline	682	102 (29.7)	100 (29.6)	0.937
Month 6	655	63 (19.2)	81 (24.9)	0.048
Anxiety (yes)				
Baseline	682	51 (14.8)	54 (16.0)	0.677
Month 6	655	29 (8.8)	47 (14.4)	0.025
Stress scores, mean (SD)				
Baseline	682	16.5 (6.8)	15.7 (6.6)	0.128
Month 6	655	12.5 (5.6)	14.9 (6.5)	< 0.001
Substance use (yes)				
Baseline	682	69 (20.1)	56 (16.6)	0.239
Month 6	655	31 (9.4)	50 (15.3)	0.021
ART non-adherence (yes)				
Baseline	682	99 (28.8)	86 (25.4)	0.959
Month 6	655	47 (14.3)	78 (23.9)	0.002

^a^Number of participants (baseline: 344, follow-up: 329)

^b^Number of participants (baseline: 338, follow-up: 326)

^c^The proportion and *p*-value is reported for comparison group who had depressive symptoms, anxiety, higher stress, substance use, and non-adherence to ART

### Effects of intervention to reduce depressive symptoms, anxiety, and stress scores at six-month follow-up

The intervention was effective in reducing the prevalence of having depressive symptoms by 56% (AOR = 0.44, 95% CI = 0.30, 0.64, *p* < 0.001) and anxiety by 46% (AOR = 0.54, 95% CI = 0.33, 0.88, *p* = 0.014) in the intervention group compared with the control group at the six-month follow-up. Also, it significantly reduced stress scores (β = − 3.98, *p* < 0.001) in the intervention group compared with the control group at the six-month follow-up. Advanced HIV-clinical stage was positively associated with the presence of depressive symptoms (AOR = 3.94, 95% CI = 1.91, 5.96, *p* = 0.001) and increased stress scores (β = 2.21, *p* = 0.001) (Table 3).

**Table 3:** GEE analyses; effects intervention to reduce depressive symptoms, anxiety, and stress scores after 6 months of follow-up

Characteristics	Depressive symptoms		Anxiety		Stress scores	
	AOR^d^(95% CI)	*P*-value	AOR^d^ (95% CI)	*P*-value	β (95% CI)	*P*-value
Baseline*Intervention^a^	0.94 (0.67, 1.33)	0.771	0.92 (0.60, 1.42)	0.600	−0.80 (− 1.76, 0.16)	0.102
Follow-up* Control^b^	0.70 (0.57, 1.08)	0.076	0.89 (0.71, 1.12)	0.329	−1.05 (− 1.52, 0.07)	0.082
Follow-up* Intervention^c^	0.44 (0.30, 0.64)	< 0.001	0.54 (0.33, 0.88)	0.014	−3.98 (− 4.94, − 3.02)	< 0.001
Gender (male)	1.25 (0.89, 1.76)	0.186	1.51 (0.99, 2.31)	0.055	0.39 (−0.55, 1.33)	0.420
Marital status (married)	0.88 (0.56, 1.37)	0.586	0.67 (0.40, 1.11)	0.120	−1.12 (−2.36, 0.12)	0.077
Age	1.00 (0.98, 1.02)	0.844	1.01 (0.99, 1.04)	0.271	−0.03 (−0.09, 0.03)	0.330
Education (≥primary)	0.87 (0.62, 1.23)	0.459	1.01 (0.66, 1.54)	0.980	0.41 (−0.40, − 0.55)	0.405
Employed/self employed	0.87 (0.60, 1.26)	0.466	0.75 (0.47, 1.19)	0.221	−0.73 (−1.74, 0.27)	0.153
HIV-clinical stage (advanced)	2.82 (1.81, 4.34)	0.001	0.96 (0.53, 1.76)	0.900	2.21 (0.93, 3.48)	0.001
Physical symptoms (present)	0.96 (0.75, 1.22)	0.759	1.25 (0.88, 1.80)	0.218	0.31 (−0.21, 0.84)	0.242

^a^Intervention participants at baseline

^b^Control participants at follow-up

^c^Intervention participants at follow-up

^d^AOR: Adjusted odds ratio

### Effects of intervention to reduce substance use at six-month follow-up

The intervention reduced the prevalence of substance use by 49% (AOR = 0.51, 95% CI = 0.31, 0.81, *p* = 0.005) in the intervention group compared with the control group. At the 6 month follow-up, men were more likely to be using substances (AOR = 1.49, 95% CI = 1.06, 2.10, *p* = 0.045) compared to women (Table 4).

**Table 4:** GEE analyses: effects of a community home-based care intervention to reduce substance use after 6 months of follow-up

Characteristics	AOR^d^ (95% CI)	*P*-value
Baseline*Intervention^a^	1.23 (0.82, 1.83)	0.209
Follow-up* Control^b^	0.88 (0.61, 1.28)	0.522
Follow-up* Intervention^c^	0.51 (0.31, 0.81)	0.005
Gender (male)	1.49 (1.06, 2.10)	0.045
Marital status (married)	0.71 (0.46, 1.08)	0.687
Age	1.00 (0.97, 1.02)	0.960
Education (≥primary)	0.87 (0.62, 1.23)	0.454
Employed/self employed	1.13 (0.79, 1.62)	0.486
HIV-clinical stage (advanced)	0.99 (0.57, 1.73)	0.997
Physical symptoms (present)	1.04 (0.63, 1.71)	0.867

^a^Intervention participants at baseline

^b^Control participants at follow-up

^c^Intervention participants at follow-up

^d^AOR: Adjusted odds ratio

### Effects of intervention to reduce non-adherence to ART at six-months follow-up

The intervention significantly reduced the non-adherence to ART by 38% (AOR = 0.62, 95% CI = 0.41, 0.95, *p* = 0.025) in the intervention group compared with the control group. Participants were more likely to be non-adherent to ART when they experienced depressive symptoms (AOR = 1.47, 95% CI = 1.04, 2.07, *p* = 0.025). Increases in stress scores were positively associated with non-adherent to ART (AOR = 1.05, 95% CI = 1.03, 1.08, *p* < 0.001).

Participants were more likely to be non-adherent to ART when they used substances (AOR = 1.73, 95% CI = 1.24, 2.41, *p* = 0.001) compared with those who did not use substances. Employed or self-employed participants were less likely to be non-adherent to ART (AOR = 0.63, 95% CI = 0.44, 0.92, *p* = 0.016) compared to employed ones (Table 5).

**Table 5:** GEE analyses: effects of intervention to reduce non-adherence to ART after 6 months of follow-up

Characteristics	AOR^d^ (95% CI)	*P*-value
Baseline*Intervention^a^	1.26 (0.88, 1.81)	0.200
Follow-up* Control^b^	1.01 (0.77, 1.33)	0.916
Follow-up* Intervention^c^	0.62 (0.41, 0.95)	0.031
Depression	1.47 (1.04, 2.07)	0.025
Anxiety	1.41 (0.94, 2.11)	0.088
Stress scores	1.05 (1.03, 1.08)	< 0.001
Substance use	1.73 (1.24, 2.41)	0.001
Gender (male)	0.83 (0.60, 1.15)	0.265
Marital status (married)	0.79 (0.52, 1.19)	0.125
Age	0.99 (0.97, 1.01)	0.710
Education (≥primary level)	0.94 (0.67, 1.31)	0.735
Employed/self employed	0.63 (0.44, 0.92)	0.016
Clinical stage (advanced)	1.45 (0.92, 2.28)	0.109
Physical symptoms	0.98 (0.69, 1.38)	0.909

^a^Intervention participants at baseline

^b^Control participants at follow-up

^c^Intervention participants at follow-up

^d^AOR: Adjusted odds ratio

## Discussion

This study revealed for the first time the positive roles of a community home-based care intervention to reduce depressive symptoms, anxiety, and stress levels of its participants after 6 months of follow-up in LMICs. The intervention was also effective in reducing the participants’ substance use and non-adherence to ART.

The community home-based care program was effective in reducing the prevalence of having depressive symptoms, anxiety and stress levels among its participants. HIV-positive people received social and emotional supports during home-visits, which may have helped them overcome their psychological problems [34, 35]. Home-based family counseling may have also improved their interpersonal relationship in family leading to their psychosocial wellbeing [36]. Moreover, they may have improved their coping skills and self-help skills when they were motivated by the home-visit to engage in self-help groups to receive psychosocial support [14, 37]. They may have developed their positive feelings when they received spiritual and religious support and counseling from the team [38].

The program was also effective in reducing substance use among its participants at the six-month follow-up. Peer support and counseling is another essential component of a community home-based care program. Peer counseling may have helped the participants to improve their coping and self-care skills as they had better trust and satisfaction towards their HIV-positive peers [39]. They may have reduced their risk of substance use when they received peer support for psychological distress risk reduction counseling for substance use [40, 41]. Also, they may have improved their health-seeking behaviors on substance use treatment when they received support from their peers at the home-visits [15]. Peer counseling also helps HIV-positive people to develop adaptive and religious coping skills which may reduce their substance use behavior [42].

The program had significant effects in reducing non-adherence to ART among its participants at the six-month follow-up. The program team carefully checked the number of consumed pills out of the number of prescribed pills. HIV-positive people may have improved their adherence when the home visit team helped their use of memory aids [43]. They also received better family support about the time of taking pills and self-care skills for the side effects of ART [44]. They received individual counseling on coping with the side effects of ART, stress, and adjustment against physical symptoms that may have helped them adhere to the treatment [45]. Seeking regular psychosocial support may have also improved ART adherence [46]. During the visits, they also may have had an opportunity to share their experiences and to learn from their peers [43, 47]. Contrary to this result, peer health workers’ support did not improve adherence to ART among HIV-positive people in Uganda [48].

This study has two main limitations. First, as participants reported their characteristics using self-reported questionnaires, the results might have been influenced by social desirability bias. To minimize this, the research team interviewed the participants in a private place. Second, the allocation of the participants to intervention and control group is also a limitation. The intervention effects might have been influenced by ongoing routine health services. Participants, however, had similar baseline characteristics for the intervention and control groups. For our analysis, we controlled the factors related to services, sociodemographics, and HIV-staging. Moreover, in selecting the participants, we selected those who were diagnosed within 5 years and those who were referred from the ART centers.

Despite these limitations, this is the first study to show the positive effects of a comprehensively designed community home-based care program on mental health and ART adherence in a resource-limited setting. Also, this study had a low attrition rate of 4%, which implies that the result might not have been much influenced by losing participants in the follow-up stage. The program is also topical and appropriate in resource-limited settings as it is peer-focused intervention.

## Conclusions

The intervention was effective in reducing depressive symptoms, anxiety, and the stress levels of its participants after the six-month follow-up. It was also effective in reducing their substance use and non-adherence to ART. As such evidence is limited in resource-limited settings, this study provided additional evidence about positive outcomes of a community home-based intervention in reducing anxiety and stress along with depressive symptoms. Additionally, as peer counseling was a unique component of this intervention, this study demonstrated that it was effective in reducing substance use among its participants. The results highlight that the program should be promoted as an essential component of care and support for HIV-positive people to address their mental health, substance use, and non-adherence in Nepal, and it should also be considered in other resource limited settings.

## Abbreviations

AIDS: Acquired immune deficiency syndrome
ART: Anti-retroviral therapy
HIV: Human immunodeficiency virus
NGO: Non-governmental organization
